# Thermo- and pH-Responsive Antimicrobial Hydrogels from Poly(2-(dimethylamino)ethyl methacrylate) and Cationic-Modified Chitosan for Sustained Papain Release

**DOI:** 10.1021/acspolymersau.5c00148

**Published:** 2025-12-10

**Authors:** Carolina Cruz Ferreira, Guilherme Frey Schutz, Iago Aguiar Dias Carmo, Lucas Novaes Teixeira, Elizabeth Ferreira Martinez, Lúcia Helena Innocentini Mei, Roniérik Pioli Vieira

**Affiliations:** 1 28132 Universidade Estadual de Campinas (UNICAMP), School of Chemical Engineering (FEQ) Albert Einstein Avenue, 500, Campinas, São Paulo 13083-852, Brazil; 2 Division of Oral Pathology, 130267 Faculdade São Leopoldo Mandic, Dr. José Rocha Junqueira Street, 13, Campinas, São Paulo 13045-755, Brazil

**Keywords:** wound, dressing, antimicrobial, chitosan, biomaterial

## Abstract

Semi-interpenetrating polymer networks (semi-IPNs) composed of poly­(2-(dimethylamino)­ethyl methacrylate) (PDMAEMA) and various concentrations of *N*-(2-hydroxypropyl)-3-trimethylammonium chitosan chloride (HTCC) (5, 10, and 25% w/w) were synthesized and evaluated as matrices for papain loading and sustained delivery. The incorporation of HTCC significantly influenced the structural, swelling, and release properties of the networks. Monomer conversion and gel fraction decreased with increasing HTCC content, reaching 91.26 and 81.36%, respectively, at 25% HTCC. Swelling studies revealed a nonlinear behavior, with the 5% HTCC sample exhibiting the highest swelling degree (458.33%), while pure PDMAEMA and the 25% HTCC formulation reached 219.78 and 168.23%, respectively. Papain release profiles, fitted to the Peppas–Sahlin model, showed diffusion-controlled kinetics, with *k*_1_ values decreasing from 31.89 h^–*m*^ (PDMAEMA) to 6.14 h^–*m*^ (PDMAEMA/HTCC25%), indicating hindered diffusion in denser networks, which may be beneficial for a more sustained therapeutic release. Antibacterial assays confirmed the potent activity of HTCC-containing formulations against *Staphylococcus aureus*, with 25% HTCC demonstrating nearly complete inhibition (∼95%), while no significant inhibition was observed against *Escherichia coli*, indicating Gram-specific selectivity. Moreover, the 25% HTCC formulation maintained fibroblast viability above 70%, further supporting these hydrogels as bioactive wound dressings combining controlled delivery with antimicrobial activity.

## Graphical abstract

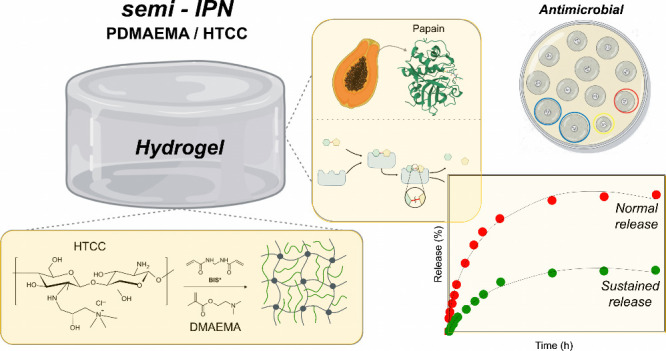

## 1 Introduction

Hydrogels are three-dimensional polymeric networks capable of absorbing and retaining large amounts of water while maintaining their structure, making them highly attractive for biomedical applications such as drug delivery, tissue engineering, and wound healing.^1,2^ Among the synthetic polymers employed in hydrogel formulation, poly­[2-(dimethylamino)­ethyl methacrylate] (PDMAEMA) has garnered significant attention due to its pH- and temperature-responsive behavior stemming from the presence of tertiary amino groups along its backbone.^3^ These functional groups allow the polymer to undergo reversible transitions in response to environmental stimuli, thereby offering tunable swelling and release characteristics ideal for controlled drug delivery systems.^4,5^

However, synthetic hydrogels based solely on PDMAEMA often lack inherent bioactivity, particularly antimicrobial properties, which are essential for applications such as wound dressings.^6,7^ To overcome this limitation, natural polymers with intrinsic antimicrobial features, such as chitosan and its derivatives, have been explored as co-components in hydrogel matrices.^8^ Chitosan is a cationic polysaccharide derived from chitin and has gained prominence due to its biocompatibility, biodegradability, and antimicrobial activity.^9–12^ When chitosan is quaternized, forming *N*-(2-hydroxypropyl) trimethylammonium chloride chitosan (HTCC), its solubility at neutral pH improves significantly, and its antibacterial efficacy increases due to the permanent positive charges on its quaternary ammonium groups.^13,14^

The combination of PDMAEMA and HTCC in a semi-interpenetrating network (semi-IPN) format can synergistically unite the smart-responsive behavior of PDMAEMA with the antibacterial and biofunctional properties of HTCC.^13^ In a semi-IPN, where one polymer is cross-linked while the other remains physically entangled to enhance mechanical and functional properties,^15,16^ the combination of responsive synthetic polymers with polysaccharide derivatives has proven to be a viable strategy, as supported by the promising results reported by Guo et al. (2007)^17^ for controlled release applications. The HTCC component not only provides bioactivity but also may enhance the network structure through physical interactions with PDMAEMA, such as ionic associations between the quaternary ammonium groups of HTCC and the protonated amines of PDMAEMA under physiological pH.

This unique network structure creates an ideal platform for the encapsulation of bioactive molecules such as papain. Papain, a proteolytic enzyme derived from *Carica papaya*,^18^ is known for its wound-healing properties, including debriding action, anti-inflammatory effects, and potential to stimulate tissue regeneration.^19–21^ However, its clinical application is often limited by instability and uncontrolled enzymatic activity in aqueous environments.^22^ Therefore, the central hypothesis of this work is that encapsulation of papain within the semi-IPN hydrogel can enhance its stability and allow for precise control over its release. This approach may not only prolong the enzyme’s therapeutic activity but also improve its efficacy, ensuring sustained delivery for optimal therapeutic outcomes.

In this study, we present a novel dual-responsive semi-interpenetrating polymer network (semi-IPN) hydrogel system based on PDMAEMA and varying concentrations of HTCC (5, 10, and 25% w/w) synthesized via radical polymerization. To our knowledge, this is the first report to combine PDMAEMA’s pH and temperature-responsive behavior with the antimicrobial functionality of HTCC in a single platform designed for controlled papain delivery. The physical and chemical properties of the resulting hydrogels were evaluated through swelling studies, gel fraction analysis, conversion efficiency, and thermal and morphological characterization. Papain release was investigated and mathematically modeled to elucidate the underlying mechanism of release. Antibacterial activity against *Staphylococcus aureus* and *Escherichia coli* was assessed to confirm the functional role of HTCC in the hydrogels. Additionally, cell viability assays were performed to establish a safe concentration threshold for HTCC for future biomedical applications. Overall, this work demonstrates that PDMAEMA/HTCC semi-IPNs are promising candidates for future *in vivo* studies as bioactive wound dressings, offering both controlled release of a wound-healing enzyme and effective antimicrobial protection.

## 2 Experimental Section

### 2.1 Chemicals

Papain (catalog number PA20221122101) was kindly supplied by Purifarma (São Paulo, Brazil). The following reagents were purchased from Sigma-Aldrich (São Paulo, Brazil): 2-(dimethylamino)­ethyl methacrylate (DMAEMA, stabilized with monomethyl ether hydroquinone, purity 98%), chitosan (CH, with a viscosimetric molecular weight of approximately 50–190 kDa and a degree of deacetylation of 83%), and glycidyl trimethylammonium chloride (GTMAC). Glacial acetic acid (ACS grade), acetone (P.A.), and ammonium persulfate (APS, P.A.) were obtained from Sinergia Científica (São Paulo, Brazil). *N*,*N*′-Methylenebis­(acrylamide) (BIS, 99%) was also acquired from Sigma-Aldrich. Prior to use, the inhibitor present in DMAEMA was removed by passage through an alumina column, while all other reagents were utilized as received without further purification.

### 2.2 Synthesis of *N*-(2-Hydroxypropyl) Trimethylammonium Chloride Chitosan (HTCC)

HTCC synthesis was carried out in a neutral aqueous environment by dispersing 3 g of low-molecular-weight chitosan (CH) in 50 mL of deionized water, maintaining the temperature at 55 °C, as illustrated in Scheme 1a. Next, a 5 mL portion of GTMAC was added, and the mixture was heated and stirred for 18 h in a reflux condenser at 55 °C. After the incubation, the resulting viscous yellow solution was centrifuged at 4000 rpm for 20 min to remove any unreacted CH. The white reaction product was isolated from the supernatant by precipitation in acetone, stirred for 24 h, and then subjected to vacuum filtration. The degree of substitution (DS) was determined through conductometric titration^23^ using a 0.1% HTCC solution, with 0.017 M AgNO_3_ as the titrant (Nova Instruments, model NI CVM). The DS was calculated using eq 1, where *V*_Ag_ and *C*_Ag_ represent the volume and concentration of AgNO_3_ at the equivalence point; *m*_HTCC_ is the mass of HTCC in the solution; *M*_GTMAC_, *M*_G_, and *M*_A_ are the molar masses of GTMAC, d-glucosamine (GlcN), and *N*-acetyl-d-glucosamine (GlcNAc), respectively; and GD denotes the average degree of deacetylation of CH.

$$\mathrm{DS}\;(\%)=\frac{V_{\mathrm{Ag}}C_{\mathrm{Ag}}}{[m_{\mathrm{HTCC}}-(V_{\mathrm{Ag}}C_{\mathrm{Ag}}M_{\mathrm{GTMAC}})/(M_{G}GD)+M_{A}(1-GD)]GD}\times 100$$ 1

**Scheme 1:**
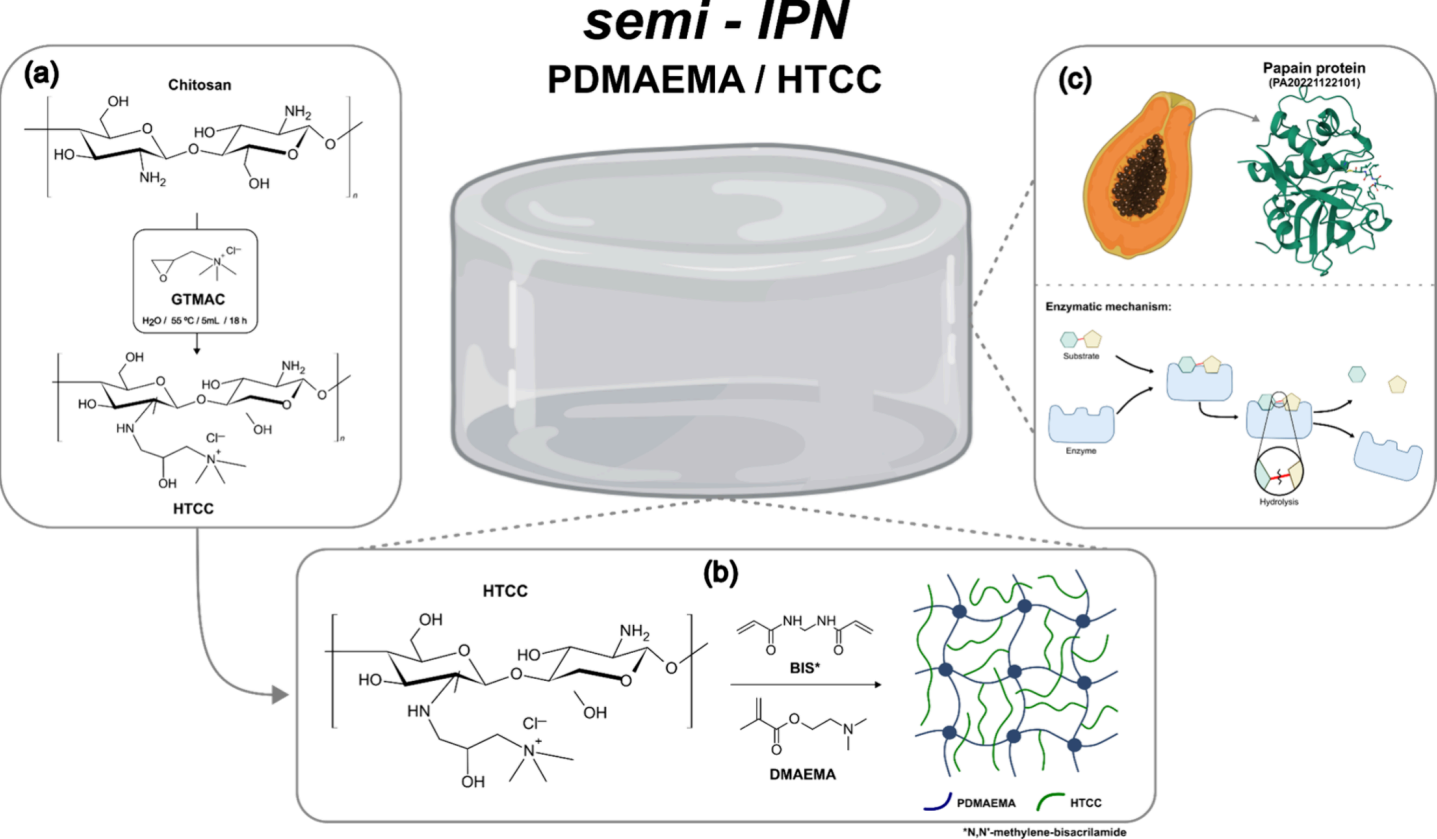
General Procedure for Synthesizing Hydrogels through the Quaternization of CH (a), Free-Radical Polymerization of DMAEMA and Crosslinking with BIS in the Presence of HTCC (b), and Incorporation of Papain (c)^a^

a This process results in the formation of semi-interpenetrating (semi-IPN) networks, where HTCC physically entangles with the PDMAEMA matrix. Created with BioRender (2025).

### 2.3 Synthesis of Poly­[2-(dimethylamino)­ethyl methacrylate]/*N*-(2-Hydroxypropyl) Trimethylammonium Chloride Chitosan (PDMAEMA/HTCC) Semi-IPN Hydrogels

The semi-interpenetrating network (semi-IPN) hydrogels were synthesized via radical polymerization of DMAEMA in the presence of HTCC utilizing ammonium persulfate (APS) as the initiator and *N*,*N*′-methylenebis­(acrylamide) (BIS) as the cross-linker, as shown in Scheme 1b. In brief, a 2% (w/v) HTCC aqueous solution was prepared and stirred magnetically until completely dissolved. Subsequently, APS was added followed by DMAEMA and BIS into 20 mL vials sealed with silicone septa. The molar ratios were 50:1 (DMAEMA/APS) and 10:1 (DMAEMA/BIS), defined after preliminary tests. The reaction mixture was purged with nitrogen for 10 min, heated to 70 °C, and stirred vigorously until the gelation process was complete. Hydrogels with 5, 10, and 25% (w/w) HTCC content were produced, along with a control sample of pure methacrylate homopolymer. The hydrogel samples were designated as PDMAEMA, PDMAEMA/HTCC5%, PDMAEMA/HTCC10%, and PDMAEMA/HTCC25%. To eliminate any unreacted CH, the synthesized hydrogels were dialyzed in deionized water for 7 days, with water replaced every 24 h. Finally, each hydrogel sample was freeze-dried using a Liobras freeze-dryer (model L101).

### 2.4 Monomer Conversion and Semi-IPN Hydrogels’ Physical Properties

The degree of conversion, which reflects the proportion of the DMAEMA monomer incorporated into the polymer network, was calculated using eq 2. In this equation, *m*_P_ denotes the final dry mass of the hydrogel, while the initial mass of the monomer (*m*_M_0__), along with the masses of the biopolymer (*m*_B_), initiator (*m*_I_), and cross-linker (*m*_R_) in the formulation, was also considered.

$$X\;(\%)=\frac{m_{P}-(m_{B}+m_{I}+m_{R})}{m_{M_{0}}}\times 100$$ 2

The gel fraction [GF(%)] measurement offers a quantitative evaluation of the efficiency of the synthesized hydrogel network. To perform this assessment, freeze-dried hydrogel samples were immersed in deionized water until they reached equilibrium swelling. Following this, the samples were freeze-dried once more, and the gel fraction was calculated by using eq 3. In this equation, *m*_f_ represents the final dry mass of the hydrogel, while *m*_0_ corresponds to its initial dry mass.

$$\mathrm{GF}\;(\%)=\frac{m_{f}}{m_{0}}\times 100$$ 3

The swelling behavior [GI(%)] of the synthesized hydrogels was assessed through a gravimetric technique. Dried hydrogel samples were placed in deionized water at 25 °C, and their masses were measured at specified time intervals. Before weighing, any excess surface water was carefully blotted off with filter paper. The degree of swelling was determined using eq 4, where *m*_t_ represents the mass of the swollen hydrogel at time *t* and *m*_0_ is the initial dry mass. The test was conducted in triplicate.

$$\mathrm{GI}\;(\%)=\frac{m_{t}-m_{0}}{m_{0}}\times 100$$ 4

Several models can be used to predict the swelling fraction (GI) profile of the hydrogels. One such model is the Peleg model,^24,25^ expressed by eq 5, which is a nonexponential, two-parameter empirical model ideal for characterizing water absorption. In this equation, *k*_1_ represents the Peleg rate constant (h%^–1^), and *k*_2_ is the Peleg capacity constant (%^–1^). Using the GI profiles obtained through eq 5, the Peleg model was fitted, and the parameters were calculated using the Statistica software.

$$\mathrm{GI}=\frac{m_{t}-m_{0}}{m_{0}}=\frac{1}{m_{0}}\left(\frac{t}{k_{1}+k_{2}t}\right)$$ 5

To evaluate the hydrogel’s pH sensitivity, dried samples were immersed in buffers at pH values of 1, 4, 7, 10, and 12 at 37 °C for 24 h, allowing them to reach equilibrium swelling. At each time point, the samples were removed; any remaining buffer was gently blotted off with filter paper, and the hydrogels were weighed. For thermosensitivity testing, the dried hydrogel samples were exposed to deionized water at temperatures of 10, 20, 30, 40, 50, and 60 °C for 24 h, also until they reached equilibrium swelling. After the excess surface water was removed with a filter paper, the hydrogels were weighed. The swelling degree for both conditions was then calculated using eq 4.

### 2.5 Morphology of the Semi-IPN Hydrogels

The surface morphology of the samples was analyzed using a scanning electron microscope (SEM, model Leo 440i, LEO Electron Microscopy/Oxford). Before imaging, the samples were sputter-coated with a thin gold layer, approximately 200 Å thick, using an EMITECH Sputter Coater (model K450).

### 2.6 Porosity of the Semi-IPN Hydrogels

The specific surface area and porosity were determined using the Brunauer–Emmett–Teller (BET) method via nitrogen adsorption utilizing a Micromeritics Gemini VII 2390 analyzer. Prior to analysis, the samples were degassed at 60 °C for 8 h using a Micromeritics VacPrep 061 to remove any adsorbed environmental substances. The BET analysis was subsequently conducted at a relative vapor pressure range of 0.01–0.3 at 77 K. The average pore diameter (*d*) was calculated using eq 6, where *V* represents the total pore volume and *A* refers to the BET surface area.

$$d=\frac{4V}{A}$$ 6

### 2.7 Fourier Transform Infrared Spectroscopy

FTIR spectroscopy was used to analyze both the synthesized HTCC and semi-IPN hydrogels. The analysis was conducted with an Agilent Technologies Cary 630 FTIR spectrometer equipped with an attenuated total reflectance (ATR) accessory containing a zinc selenide (ZnSe) crystal. The spectral range extended from 4000 to 650 cm^–1^, with a resolution of 4 cm^–1^ and 32 scans taken per measurement.

### 2.8 Thermogravimetric Analysis

The thermal stability of the synthesized HTCC and semi-IPN hydrogels was assessed through thermogravimetric analysis (TGA) under an inert nitrogen atmosphere (flow rate of 50 mL min^–1^). The samples were heated from 25 to 600 °C at a rate of 10 °C min^–1^. The analysis was performed using a Shimadzu TGA-50 M thermal analyzer coupled with a Mettler-Toledo MX5 microanalytical balance.

### 2.9 Antibacterial Activity

The antibacterial effectiveness of the hydrogels was assessed using the colony-forming unit (CFU) counting method following a procedure analogous to our previous study.^26^ Briefly, the semi-IPN hydrogels were tested against standard strains of *Escherichia coli* (ATCC 25922) and *Staphylococcus aureus* (ATCC 25923), both obtained from the American Type Culture Collection (ATCC) and maintained at the Microbiology Laboratory of Faculdade São Leopoldo Mandic. The bacterial strains were thawed and cultured in a brain heart infusion (BHI) medium (Himedia, India) at 37 °C under microaerophilic conditions (TECNAL, model TE399) for 24 h. An aliquot was transferred to a Petri dish with BHI agar to promote colony growth. Broth cultures were prepared to a final density of 3.0 × 10^8^ cells mL^–1^ (McFarland standard no. 1), confirmed using a spectrophotometer (520 nm, Epoch, Bio-Tek). The hydrogels were sterilized under UV light for 15 min, hydrated in sterile BHI solution, and inoculated with either *E. coli* or *S. aureus* for 24 h. After vortexing for 30 s, the samples were serially diluted and plated onto BHI agar plates in triplicate. The plates were incubated at 37 °C for 24 h, and colony counting was done manually. Results were expressed as the reduction in CFU count.

### 2.10 *In Vitro* Cytotoxicity

The *in vitro* cytotoxicity of the semi-IPN hydrogels was evaluated following the method used in our previous study.^26^ Briefly, murine fibroblast cells from the NIH/3T3 line (CRL-1658), sourced from ATCC, were thawed and placed in centrifuge tubes with 5 mL of Dulbecco’s modified Eagle’s medium (DMEM; Sigma). After centrifugation at 336*g* for 3 min, the supernatant was discarded. The cells were cultured in 25 cm^2^ flasks (Sarstedt) containing DMEM supplemented with 10% fetal bovine serum (Nutricell), 100 IU mL^–1^ penicillin (Sigma), and 50 μg mL^–1^ streptomycin (Sigma). The medium was refreshed every 2 days, and cell growth was monitored using an inverted phase microscope (Nikon Eclipse TS100). Cells were maintained at 37 °C under a humidified 5% CO_2_ atmosphere. For the experiment, 3T3 cells were seeded in 96-well plates at a density of 110 cells mm^–2^. After 24 h, the cells were exposed to the hydrogels in accordance with ISO 10993 guidelines. Cell viability was assessed using the MTT assay (Mosmann, 1983). MTT solutions (5 mg mL^–1^ in phosphate-buffered saline, PBS) were prepared, and the cultures were incubated with a 10% MTT solution for 4 h at 37 °C in a 5% CO_2_ atmosphere. Following incubation, the cultures were washed with 200 μL of warm PBS. Then, 150 μL of dimethyl sulfoxide (DMSO) was added to each well and agitated for 5 min to dissolve the precipitate. Aliquots of 100 μL were transferred to a new plate for absorbance measurement using a spectrophotometer (570 nm; Epoch). Cell viability was determined from the optical density with results expressed as absorbance.

### 2.11 Incorporation and *In Vitro* Release of Papain

The papain was incorporated using an adsorption method, where the dried hydrogels were immersed in a 200 mg mL^–1^ aqueous papain solution until equilibrium was reached, as depicted in Scheme 1c. The hydrogels were then freeze-dried again. The mass of incorporated papain (*m*_PP_) was calculated using eq 7, in which *V*_0_ and *C*_0_ represent the volume and concentration of the initial papain solution and *V*_1_ and *C*_1_ correspond to the volume and concentration of the solution after the equilibrium of adsorption at 25 °C.

$$m_{\mathrm{PP}}=V_{0}C_{0}-V_{1}C_{1}$$ 7

The dried papain-loaded hydrogels were subsequently immersed in PBS (pH 7.4) at 37 °C under stirring to begin the *in vitro* release determination. At specific time intervals, an aliquot of the release medium was analyzed by using a Thermo Genesys 6 UV–vis spectrometer, and an equal volume of fresh, preheated PBS (37 °C) was added to replace it. The papain concentration in the release medium was measured at 278 nm based on a previously established calibration curve. For subsequent aliquots, values were adjusted by using the dilution correction factor. The cumulative percentage of papain released was then calculated using eq 8, where *V*_L_ represents the total volume of the release medium, *C*_*n*_ is the concentration after the *n*th sample, *V*_A_ is the volume of the withdrawn aliquot, *M*_*t*_ is the mass of papain at time “*t*”, and *M*_∞_ is the mass of papain at the equilibrium.

$$\frac{M_{t}}{M_{\infty }}=\frac{V_{L}C_{n}-V_{A}\sum_{1}^{n-1}C_{i}}{m_{\mathrm{PP}}}\times 100$$ 8

The cumulative release data acquired from the experiments as a function of time (h) were subjected to mathematical model fitting using Statistica 10, obtaining insights into the release mechanism. Two classical mathematical models were used in this procedure. The first one was the Korsmeyer–Peppas model, represented by eq 9. This model offers an empirical description from controlled-release dosage forms such as slabs, spheres, cylinders, or discs^27^ but is usually more appropriate to nonswellable matrices.

$$\frac{M_{t}}{M_{\infty }}=k\cdot t^{n}$$ 9

in which *k* is the Korsmeyer–Peppas kinetic constant (h^–*n*^) related to the characteristics of the macromolecular network system and the substance to be released, and *n* is the diffusional exponent for substance release, which is characteristic of the mechanism. It is important to note that eq 9 was used to analyze the first 60% of the release curve.

The Peppas–Sahlin model (eq 10) was also applied to the experimental data due to its ability to account for both diffusional and relaxational contributions within release mechanisms.^28^

$$\frac{M_{t}}{M_{\infty }}=k_{1}\cdot t^{m}+k_{2}\cdot t^{2m}$$ 10

in which *k*_1_ and *k*_2_ are the kinetic constants (h^–*m*^ and h^–2*m*^). The term *k*_1_·*t* ^*m*^ denotes the Fickian contribution, while the second term on the right side delineates the Case-II relaxational contribution. The coefficient *m* is the purely Fickian diffusion exponent for a device of any geometrical shape.

### 2.12 Statistical Analysis

Experimental results were obtained in triplicate and are presented as mean ± standard deviation. Data were analyzed using analysis of variance (ANOVA) followed by the least significant difference (LSD) test. When significant differences were detected, the Tukey HSD multiple comparison test was applied. A significance level of 5% (*p* < 0.05) was considered for all statistical analyses.

## 3 Results and Discussion

### 3.1 Synthesis and Characterization of *N*-(2-Hydroxypropyl) Trimethylammonium Chloride Chitosan (HTCC)

Figure 1a presents the FTIR spectra of chitosan (CH) before and after modification via the reaction with GTMAC (HTCC). The CH spectrum exhibits a broad band around 3000 cm^–1^, attributed to the overlapping stretching vibrations of hydroxyl (−OH) and amine (−NH_2_) groups, indicative of hydrogen bonding.^29,30^ The asymmetric and symmetric stretching vibrations of the aliphatic −CH and −CH_2_ groups appear at 2922 and ∼2870 cm^–1^, respectively. A distinct signal at 1652 cm^–1^ corresponds to the carbonyl (C=O) stretching of residual *N*-acetyl groups, while the amide band, observed at 1595 cm^–1^, arises from N–H bending and C–N stretching, characteristic of primary amine groups (−NH_2_).^26^ The spectrum also reveals C–H bending vibrations of methyl (−CH_3_) and methylene (−CH_2_) groups at 1420 and 1378 cm^–1^. Additionally, the C–N stretching band associated with amide and amine functionalities appeared at 1324 cm^–1^. Vibrations from glycosidic linkages (C–O–C) are identified at 1155 and 1069 cm^–1^, corresponding to the polysaccharide backbone, while the C–O stretching of primary alcohols (−OH) at C3 and C6 positions is detected around 1030 cm^–1^.^31,32^

**Figure 1:**
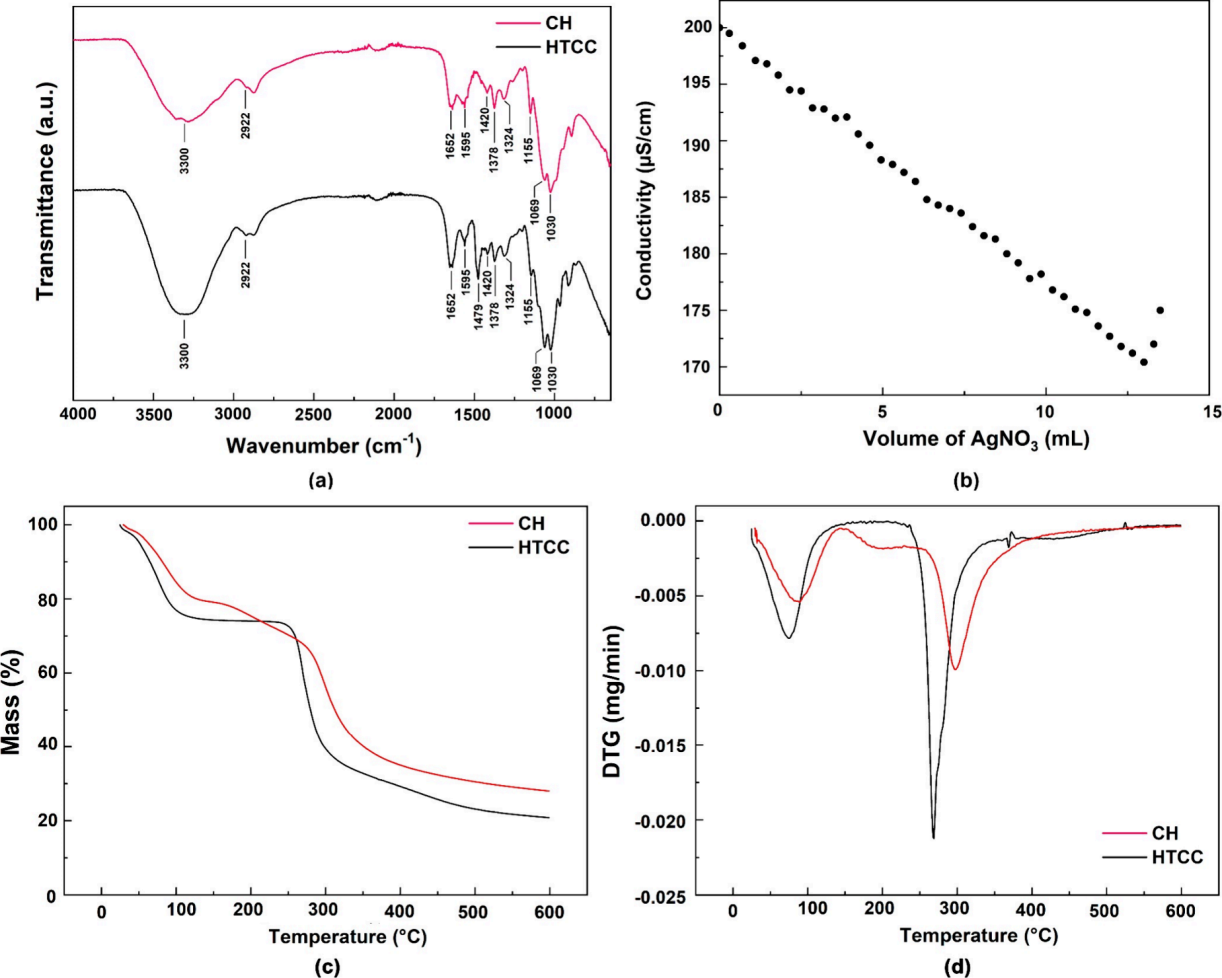
(a) FTIR spectra of CH and HTCC, (b) conductometric titration curve of HTCC, and (c, d) TGA/DTG curves of CH and HTCC.

HTCC exhibited similar peaks, with some signal modifications characteristic of the functional group incorporated into the CH backbone. The band at 3300 cm^–1^ showed increased intensity upon functionalization, which may be associated with higher water absorption resulting from HTCC’s greater hydrophilicity compared to CH. Additionally, the peak at 1652 cm^–1^ intensified, indicating the modification of primary amines of CH due to their reaction with GTMAC. Reports from the literature indicated that this reaction occurs via nucleophilic substitution of NH_2_ from epoxy groups, resulting in secondary amines.^23,33–35^ More importantly, reaction confirmation is supported by the appearance of a new peak at 1479 cm^–1^, attributed to ammonium methyl groups [−N^+^(CH_3_)_3_].^36^ The band around 1030 cm^–1^ remained unchanged, suggesting no hydroxyl substitution at C3 and C6 in the amine structure.^37^ Finally, the degree of NH_2_ substitution in the reaction was estimated at 68% using eq 1. The quantification of Ag^+^ in the solution was determined via conductometric titration based on the calibration curve shown in Figure 1b.

The thermogravimetric analysis (Figure 1c,d) compares the thermal stability of CH and HTCC and is an additional indicator of successful chemical reaction. Both materials exhibit an initial mass loss below 100 °C primarily due to the evaporation of adsorbed water, which is more pronounced in HTCC due to its higher hydrophilicity, as previously associated with specific intense bands in the FTIR spectrum. The major degradation event occurred between 200 and 400 °C, associated with the depolymerization and decomposition of the polysaccharide backbone.^38^ Notably, HTCC exhibited an earlier onset of thermal degradation compared with CH (Table 1), suggesting that functionalization with quaternary ammonium groups reduced its thermal stability. This effect can be attributed to the disruption of CH’s intermolecular hydrogen bonds, leading to increased polymer chain mobility and lower thermal resistance.^38,39^ Furthermore, HTCC showed a higher mass loss in the later stages of degradation, indicating a greater extent of decomposition. The lower residual mass at 600 °C for HTCC compared with CH further supports this trend, suggesting that the introduction of quaternary ammonium groups enhanced thermal degradation.

**Table 1:** Thermal Decomposition Parameters of CH and HTCC, including *T*_onset_, *T*_max_, Mass Loss, *T*_50_%, and Residual Mass

sample	degradation stage	*T*_onset_ (°C)	*T*_max_ (°C)	mass loss (%)	*T*_50%_(°C)	residue (%)
CH	I	41.43	86.67	12.54	312.04	27.75
	II	264.19	297.68	40.61		
HTCC	I	39.15	74.08	25.01	278.86	20.63
	II	242.66	268.98	40.72		

### 3.2 Synthesis and Physical Properties of PDMAEMA/HTCC Semi-IPN Hydrogels

The monomer (PDMAEMA) conversion and gel fraction results are presented in Table 2, where the lowest values for both parameters were observed in the presence of 25% HTCC. This reduction can be attributed to the long polymeric chains of HTCC, which likely hinder the mobility of free radicals during the initiation step, limiting their ability to effectively reach other monomers and propagate the polymerization process. As a result, the decreased propagation efficiency leads to a lower monomer conversion and gel fraction, as HTCC may also contribute to the formation of a more loosely bonded polymeric matrix. The Peleg model is widely applied to describe the swelling behavior of hydrogels, particularly for characterizing the kinetics of water uptake and swelling. Figure 1a shows the fitted Peleg model for the different hydrogel formulations, demonstrating a strong correlation between the predicted values and experimental data. The corresponding Peleg model parameters are listed in Table 2.

**Table 2:** Monomer Conversion of DMAEMA into the Hydrogel, Gel Fraction, Equilibrium Swelling Degree, Peleg Model Parameters Describing Swelling Kinetics, and Determination Coefficients (*R* ^2^) Evaluating the Model’s Fit Accuracy

sample	monomer conversion (%)	gel fraction (%)	equilibrium swelling degree (%)	*k*_1_ (Peleg parameter)	*k*_2_ (Peleg parameter)	***R*** **^2^**
PDMAEMA	96.961 ± 0.384^a^	93.853 ± 0.311^a^	219.784 ± 0.124^a^	1.6941 ± 0.0237	0.4214 ± 0.0194	0.9884
PDMAEMA/HTCC5%	95.463 ± 0.598^b^	82.497 ± 0.224^b^	458.331 ± 0.749^c^	1.5953 ± 0.0406	0.2008 ± 0.0337	0.9907
PDMAEMA/HTCC10%	93.928 ± 0.673^b^	91.018 ± 0.395^c^	385.664 ± 0.492^b^	2.4443 ± 0.0945	0.2243 ± 0.0418	0.9845
PDMAEMA/HTCC25%	91.257 ± 0.482^a^	81.357 ± 0.264^a^	168.234 ± 0.586^b^	4.3394 ± 0.0754	0.5475 ± 0.0362	0.9903

Different letters indicates statistical difference (p < 0.05).

The swelling profiles of the synthesized hydrogels after immersion in deionized water at 25 °C are presented in Figure 2a. It can be observed that all samples absorbed water immediately upon contact, with the highest retention occurring within the first few minutes (<5 min), reaching equilibrium within 60 min. All samples exhibited a swelling degree greater than 100%, and the pictures in Figure 2b illustrate the difference among the samples before and after equilibrium swelling. It is noted that the swelling degree increased with the incorporation of HTCC at low concentrations. However, as the HTCC fraction increases, the material’s swelling capacity decreases, with the PDMAEMA/HTCC25% composition exhibiting lower hydration compared to the pure PDMAEMA hydrogel. The presence of quaternary ammonium and hydroxyl groups in HTCC likely contributes to an increased swelling at lower concentrations. Being more hydrophilic than PDMAEMA, HTCC facilitates the penetration of water molecules into the polymer network, enhancing the water uptake and swelling capacity. On the other hand, at higher concentrations, the formation of a more entangled and compact network can restrict water diffusion, reducing the hydrogel’s swelling ability. This denser structure limits the availability of free spaces within the polymer matrix, thereby decreasing the overall water absorption capacity.^40^

**Figure 2:**
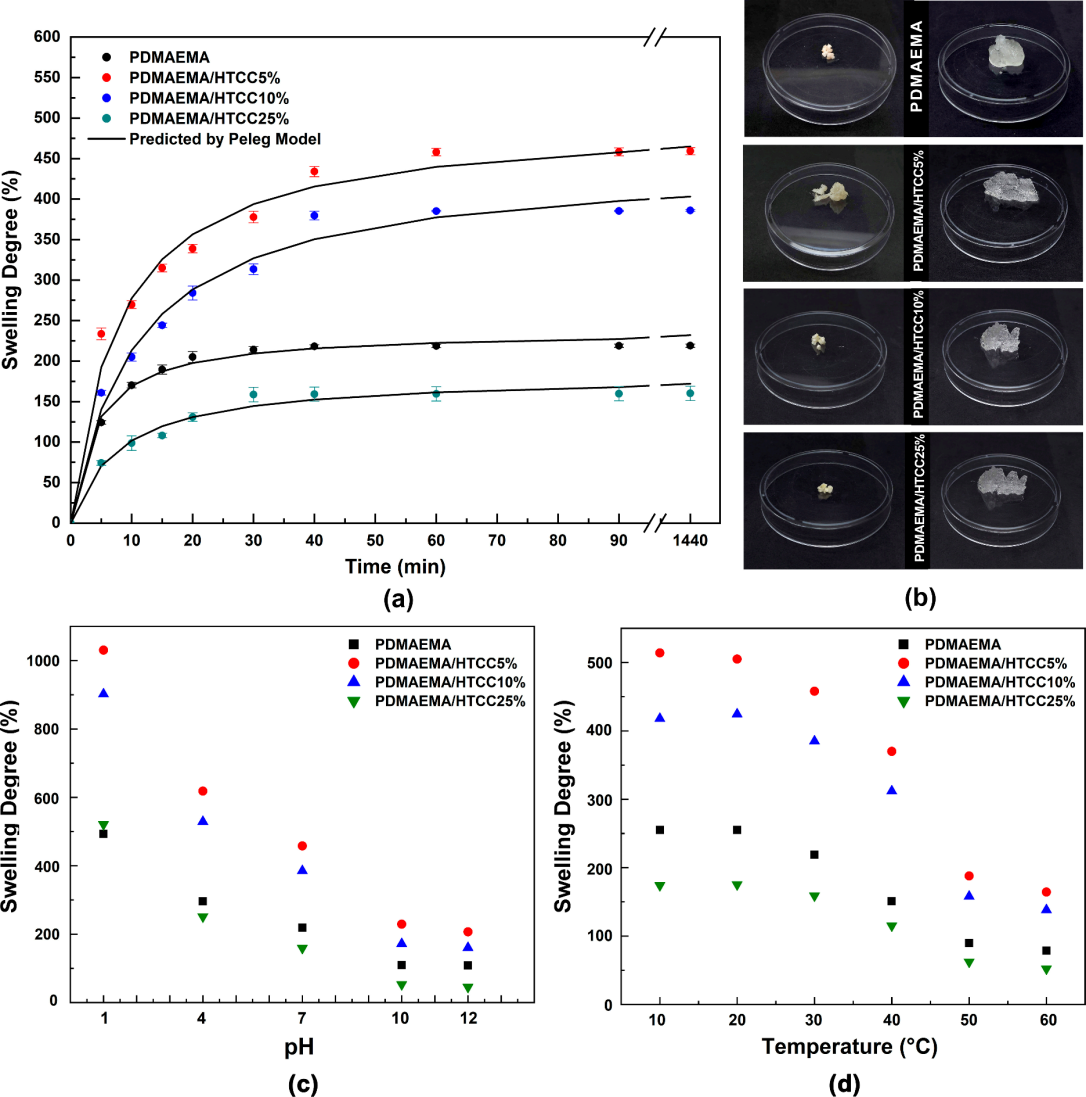
(a) Swelling kinetics of the hydrogels with Peleg model fitting, (b) images of hydrogels before and after 24 h immersion in deionized water, (c) equilibrium swelling of semi-IPN hydrogels at different pH values (1–12), and (d) influence of the temperature on equilibrium swelling degree.

The results obtained for hydrogels containing 5 and 10% HTCC are consistent with the findings reported by Wei et al. (2017)^16^ for salecan-PDMAEMA semi-IPN hydrogels. In their study, increased polysaccharide incorporation led to higher swelling ratios compared to pure PDMAEMA, which were attributed to the hydrophilic nature of the polysaccharide. This balance between polymer composition and cross-linking plays a critical role in modulating the hydrogel’s swelling behavior.

PDMAEMA is well-known for its dual responsiveness (pH and temperature), and this behavior remained unchanged in the presence of HTCC. The pH responsiveness of the hydrogels was evaluated in various buffer solutions, and the results are shown in Figure 2c. A pronounced swelling was observed under acidic conditions, with the degree of swelling significantly decreasing as the pH increased. This pH-dependent behavior can be attributed to the presence of tertiary amine side groups in the PDMAEMA polymer chains, which become protonated in acidic environments, imparting a cationic charge.^41^ PDMAEMA’s ability to undergo protonation and deprotonation in response to pH changes is a key feature of its behavior. In acidic conditions, the protonation of the tertiary amine groups induces electrostatic repulsion between the polymer chains carrying quaternary ammonium groups, leading to an expansion of the gel network and an increase in swelling. As the pH exceeds 8, deprotonation of the amine groups occurs, eliminating the electrostatic repulsion.^3,4^ This unique pH-responsive nature of PDMAEMA is critical in various applications such as drug delivery systems, where controlled release and responsiveness to environmental changes are essential.

Moreover, Figure 2d illustrates the temperature responsiveness of the synthesized PDMAEMA/HTCC-based hydrogels, clearly showing that the incorporation of HTCC does not interfere with the thermoresponsive behavior of pure PDMAEMA, potentially broadening the range of applications of the synthesized semi-IPN hydrogels. Overall, the swelling capacity of the hydrogels decreases as the temperature increases due to the conformational collapse of PDMAEMA above its LCST (lower critical solution temperature). This transition occurs because monomer–monomer interactions become more dominant than monomer–solvent interactions, leading to reduced water retention and causing the hydrogel to shrink.^4^

### 3.3 Morphology and Porosity of the PDMAEMA/HTCC Semi-IPN Hydrogels

Figure 3 presents SEM images of the morphological structure of PDMAEMA-based hydrogels after freeze-drying, revealing a well-defined, evenly spaced porous network. These open pores facilitate fluid penetration into the gel, directly influencing the swelling behavior of the hydrogels. The 3D porous architecture plays a crucial role in exudate absorption, oxygen exchange, and maintaining a moist wound environment, all of which are essential for effective healing. This porosity not only supports a hydrated environment conducive to cellular survival, adhesion, and migration but also provides an extracellular matrix-like framework that enhances tissue regeneration and accelerates the wound-healing process.^42^

**Figure 3:**
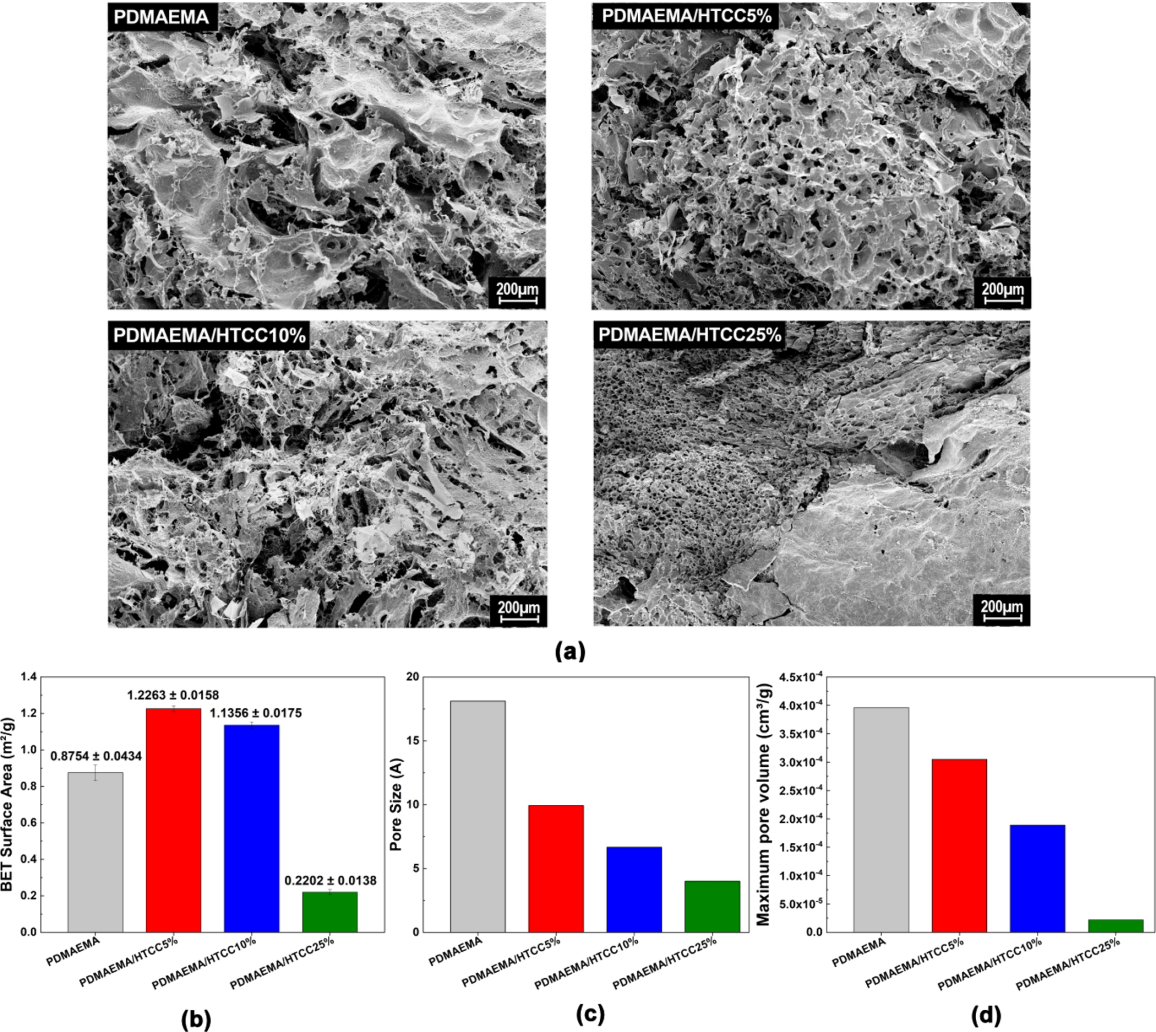
(a) SEM images of freeze-dried semi-IPN hydrogel samples, (b) specific surface area, (c) pore size distribution, and (d) maximum pore volume, all determined by the BET technique.

The increasing incorporation of HTCC leads to a more compact hydrogel network, as evidenced by SEM micrographs and pore size distribution analysis, with the most pronounced densification observed at 25% HTCC. This structural compaction directly correlates with the reduced water uptake capacity of this formulation, as shown in Figure 2a. While other formulations also exhibited a decrease in pore size, their higher surface area suggests a more interconnected porous network, which may compensate for the reduction in individual pore dimensions by facilitating fluid transport. In contrast, the HTCC25% hydrogel displays a significant decline in equilibrium swelling, likely due to strong interactions between CH hydroxyl groups and PDMAEMA tertiary amines.^43,44^ These interactions reduce the availability of active sites for water binding, limiting the formation of ice crystals during the freezing process and resulting in smaller pores. This structural modification not only impacts the swelling behavior but may also influence the mechanical properties and overall functionality of the hydrogel, highlighting the delicate balance among porosity, fluid absorption, and network stability.

### 3.4 Fourier Transform Infrared Spectroscopy (FTIR) of PDMAEMA/HTCC Semi-IPN Hydrogels

The FTIR spectrum of the PDMAEMA hydrogel exhibited characteristic bands corresponding to its functional groups (Figure 4). Signals at 2946, 2821, and 2771 cm^–1^ are attributed to the C–H stretching vibrations of aliphatic −CH_2_ and −CH_3_ groups, including those in the dimethylamino (−N­(CH_3_)_2_) moiety.^4^ The strong band at 1724 cm^–1^ corresponds to the C=O stretching vibration of the ester carbonyl group in the methacrylate backbone.^4,45^ The peaks at 1455 and 1386 cm^–1^ are associated with the bending vibrations of the −CH_2_ and −CH_3_ groups, with the latter linked to the symmetric bending of the dimethylamino group. The absorption at 1233 cm^–1^ arises from the C–O–C stretching vibrations of the ester functionality, while the band at 1145 cm^–1^ corresponds to asymmetric C–O stretching.^4,46^

**Figure 4:**
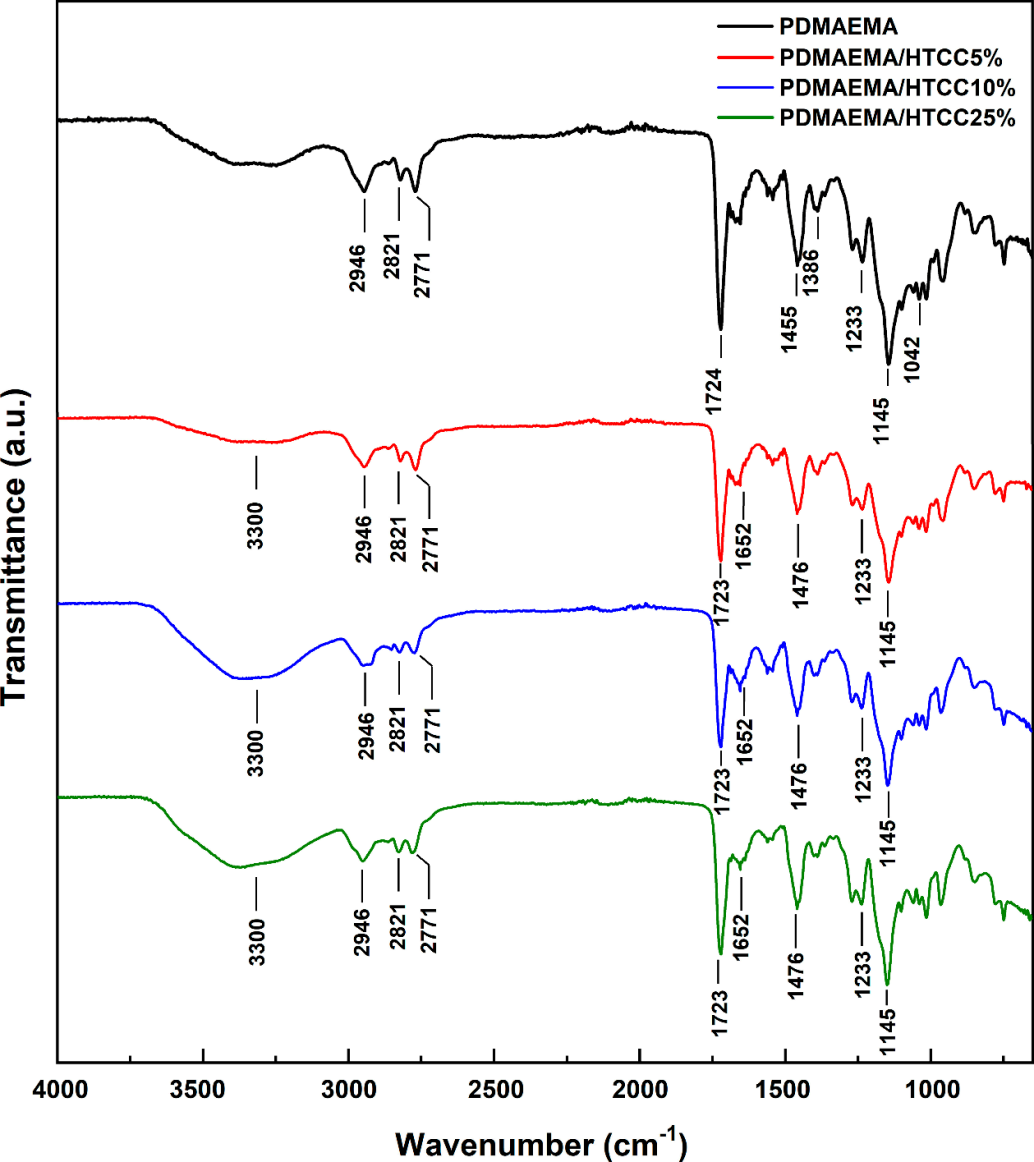
FTIR spectra of PDMAEMA and semi-IPN hydrogels containing HTCC at 5, 10, and 25% concentrations.

Finally, the peak at 1042 cm^–1^ is attributed to the C–N stretching of the tertiary amine, confirming the presence of pH-responsive functional groups in the pristine PDMAEMA hydrogel.^4^ The incorporation of different amounts of HTCC into PDMAEMA-based hydrogels altered the FTIR spectrum by shifting existing peaks due to electrostatic interactions and hydrogen bonding. The absorption bands observed at 1640 and 1480 cm^–1^ can be attributed to the C=O stretching vibration of the secondary amide and the C–H bending of the trimethylammonium groups, respectively.^23,47^ The C–N stretching at 1042 cm^–1^ shifted due to interactions between PDMAEMA’s tertiary amines and HTCC’s quaternary ammonium groups. Additionally, the 3200–3500 cm^–1^ region exhibited a broader O–H/N–H stretching band, which intensified with increasing HTCC content. As the HTCC concentration increased, these spectral changes became more pronounced, which may also be associated with the higher moisture retention in formulations containing HTCC.

### 3.5 Thermogravimetric Analysis of the PDMAEMA/HTCC Semi-IPN Hydrogels

The TGA and DTG curves, representing the thermal decomposition behavior of the hydrogels, are shown in Figure 5. As observed, the decomposition process occurred in three major stages, influenced by the presence and ratio of both HTCC and the PDMAEMA structures. The key thermogravimetric parameters extracted from the thermograms are summarized in Table 3. According to the thermograms in Figure 5, all semi-IPN hydrogels exhibited an initial minor weight loss around 100 °C, which was attributed to the evaporation of bound water and surface moisture. This event also includes the release of physically and chemically bound water molecules, more evident in HTCC-rich samples due to the hydrophilicity of quaternary ammonium and hydroxyl groups.

**Figure 5:**
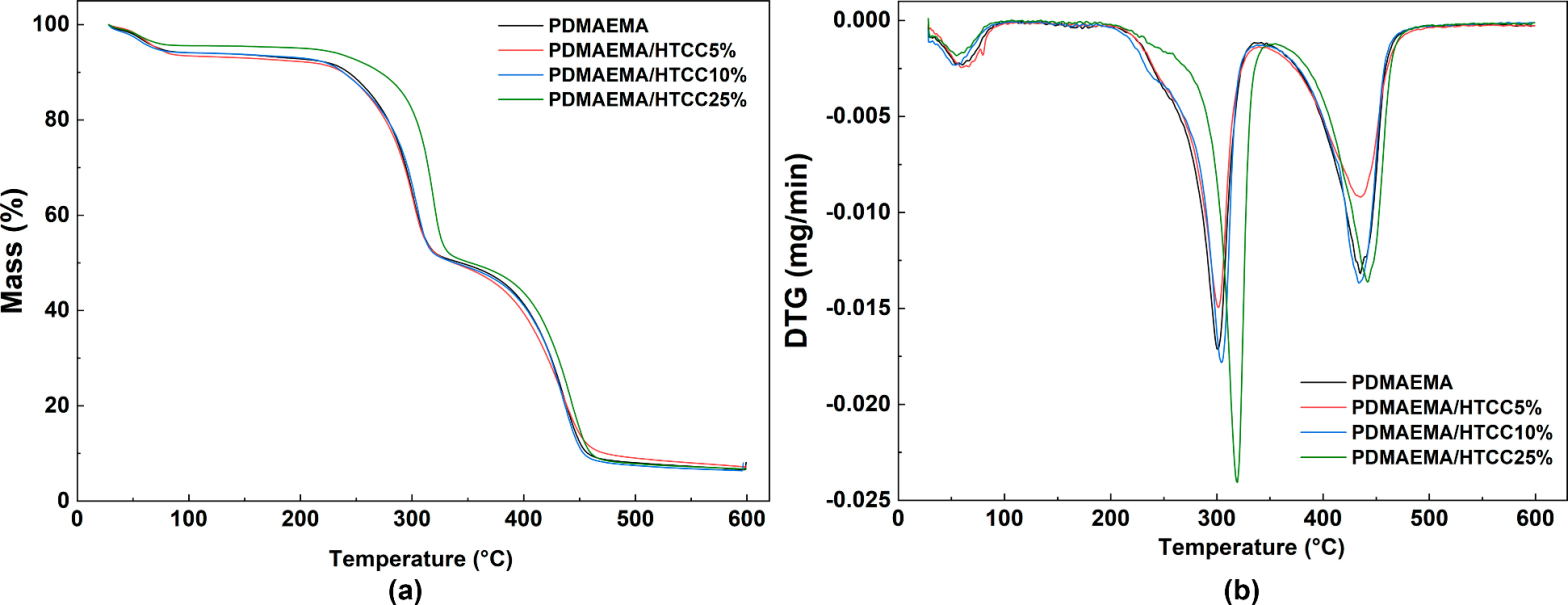
(a) TGA curve and (b) DTG illustrating the thermal degradation stages for the semi-IPN hydrogels, including the onset temperature and peak decomposition rate.

**Table 3:** Main Thermal Decomposition Parameters of the Samples, including *T*_onset_, *T*_max_, Mass Loss (%), *T*_50_%, and Residual Mass

sample	degradation stage	*T*_onset_ (°C)	*T*_max_ (°C)	mass loss (%)	*T*_50%_(°C)	residue (%)
PDMAEMA	I	37.47	55.91	4.54	343.43	8.14
	II	226.72	300.56	40.61		
	III	364.52	435.01	39.26		
PDMAEMA/HTCC5%	I	34.96	55.99	16.03	332.52	7.09
	II	248.85	285.54	45.60		
	III	396.05	429.00	31.18		
PDMAEMA/HTCC10%	I	50.61	83.48	16.77	341.26	7.99
	II	239.15	266.00	45.76		
	III	385.63	419.61	29.22		
PDMAEMA/HTCC25%	I	49.99	81.42	18.29	351.61	6.74
	II	239.88	260.69	47.61		
	III	388.01	421.70	26.10		

In the first stage of macromolecular thermal degradation, the onset temperature (*T*_onset_) for semi-IPN hydrogels is approximately 300 °C across all compositions, with a weight loss ranging from 39.33 to 42.32%. This stage is primarily associated with the cleavage of ester linkages in PDMAEMA macromolecular chains and the degradation of HTCC. During this step, the breakdown of chitosan (CH) and HTCC backbones generates volatile fragments, such as CO_2_, CO, ammonia, and low-molecular-weight aliphatic compounds derived from glycosidic and amide bond scission. The final degradation stage, resulting in weight losses between 43.15 and 44.85%, corresponds to the decomposition of the polymer backbones. This process leads to the release of saturated or unsaturated aliphatic fragments of higher molecular weight, along with CO, CO_2_, carboxylic acids, and ketones, which may contribute to the formation of aromatic structures.^48^ At approximately 500 °C, the remaining residue consists mainly of thermally stable carbonaceous char and minor inorganic salts originating from the chitosan backbone, confirming the complete degradation of the organic components.

A slight decrease in the thermal stability of the PDMAEMA/HTCC semi-IPN hydrogels was observed, as evidenced by the reduction in *T*_max_ during the second thermal event, which corresponds to the stage of maximum mass loss. This behavior suggests that the formation of a second network, from the HTCC, has influenced the degradation mechanism of the material. The presence of HTCC likely introduced additional interactions such as hydrogen bonding and electrostatic interactions, which could disrupt the regular polymeric structure of PDMAEMA. As a result, the thermal decomposition pathway may have been altered, leading to the earlier onset of thermal degradation and lower *T*_max_. Similar effects have been reported in other semi-IPN systems, where the introduction of a secondary network was shown to promote chain scission at lower temperatures. Specifically, in PDMAEMA/carboxymethyl starch hydrogels, Nita et al. (2020) observed a decrease in thermal stability attributed to the rearrangement of intra- and intermolecular bonds, which facilitated the breakdown of the polymer backbone.^49^

In the case of PDMAEMA/HTCC hydrogels of this present study, the electrostatic interactions between the quaternary ammonium groups of HTCC and the tertiary amine groups of PDMAEMA, along with potential hydrogen bonds between the hydroxyl groups of HTCC and the ester carbonyl groups of PDMAEMA, likely contributed to a less stable polymer network. This disruption of the polymer structure may enhance the material’s susceptibility to thermal degradation, leading to an earlier onset of chain scission and mass loss at lower temperatures compared to pure PDMAEMA hydrogels. The DTG curve of the PDMAEMA/HTCC25% sample (Figure 5b) shows a distinct profile with broader and overlapping thermal events compared to the other formulations. This behavior is related to the higher densification of the polymeric network at 25% HTCC, as confirmed by the morphological analysis (Section 3.3), which revealed a more compact structure. Increased compactness, driven by stronger ionic and hydrogen bonding interactions, modifies the decomposition pathway, resulting in merged and broadened degradation peaks. The decomposition parameters listed in Table 3, such as onset and peak temperatures, were calculated from these overlapping transitions and remain consistent with the overall trend that the presence of HTCC influences the material’s degradation mechanism.

### 3.6 *In Vitro* Antibacterial and Cytotoxicity of the PDMAEMA/HTCC Semi-IPN Hydrogels

The antibacterial activity of CH and its quaternary derivatives, such as HTCC, has shown some variability across different studies. For example, *N*,*N*,*N*-diethylmethyl-CH exhibits enhanced antibacterial activity against *E. coli* compared to native CH, with the activity increasing under acidic conditions.^50^ HTCC, prepared by reacting CH with GTMAC, has demonstrated superior antibacterial efficiency against both *E. coli* and *S. aureus* compared to CH.^34,51^ In contrast, Chi et al.^52^ found no significant antibacterial activity of HTCC against *E. coli*, while Qin et al.^53^ reported that HTCC’s antibacterial activity was stronger under alkaline conditions than under weak acidic conditions. These discrepancies suggest that the antibacterial effectiveness of HTCC may be influenced by various factors, including the preparation method, concentration, pH conditions, and bacterial strain.

The activity against *S. aureus* and *E. coli* of PDMAEMA/HTCC-based hydrogels is demonstrated in Figure 6a. The positive control exhibited complete bacterial inhibition, as expected. For the semi-IPN hydrogel samples, the CFU reduction for *S. aureus* increased with the HTCC content, indicating a dose-dependent effect. The PDMAEMA/HTCC25% sample achieved the highest activity against the *S. aureus* strain, nearly matching the positive control. Conversely, the 5% HTCC sample shows a moderate reduction, suggesting an insufficient HTCC concentration for complete bacterial inhibition. The trend confirms that higher HTCC incorporation enhances antibacterial properties, likely due to increased electrostatic interactions between the cationic polymer and bacterial membranes.^54^ The absence of detectable antibacterial activity against *E. coli* in Figure 6a could be attributed to differences in the bacterial cell wall structure. Unlike *S. aureus*, which is Gram-positive and has a thick peptidoglycan layer with a high density of anionic teichoic acids,^55^ *E. coli* is a Gram-negative bacterium with an outer membrane composed of lipopolysaccharides. This outer membrane acts as a protective barrier, reducing the penetration of cationic antimicrobial agents such as HTCC. Additionally, *E. coli* possesses more efficient efflux pumps and enzymatic defense mechanisms that may further reduce the effectiveness of the antimicrobial hydrogel.^56^

**Figure 6:**
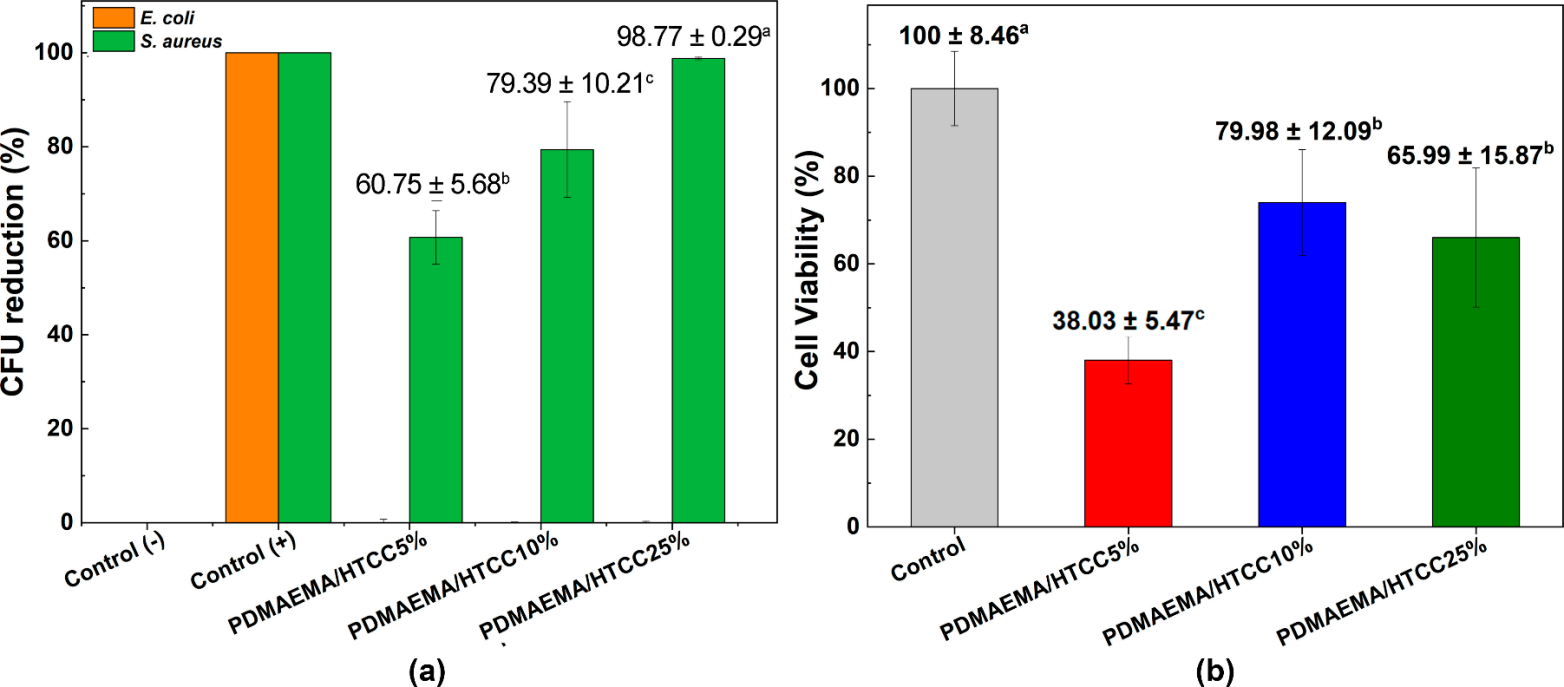
(a) Antimicrobial activity against reference strains of *Escherichia coli* (ATCC 25922) and *Staphylococcus aureus* (ATCC 25923) and (b) cytotoxicity assessment using NIH/3T3 murine fibroblast cells.

Figure 6b illustrates the cytotoxicity of the semi-IPN hydrogels against murine fibroblasts, assessed via an MTT assay. Compared to the control, all HTCC-containing samples exhibited a significant reduction in cell viability. PDMAEMA/HTCC5% showed the greatest decrease in cell viability (38.03 ± 5.47), indicating the highest cytotoxicity, while PDMAEMA/HTCC10% and PDMAEMA/HTCC25% displayed intermediate values (79.98 ± 12.09 and 65.99 ± 15.87, respectively). The higher cytotoxicity of the PDMAEMA/HTCC5% sample suggests that it may be more aggressive toward mammalian cells. Since CH-based materials are well-known for improving the biocompatibility of hydrogels,^57–59^ the higher cytotoxicity of this sample may be attributed to differences in HTCC distribution or interaction at lower concentrations. In contrast, PDMAEMA/HTCC10% and PDMAEMA/HTCC25% maintained antibacterial efficacy while exhibiting lower cytotoxicity than PDMAEMA/HTCC5%. Notably, PDMAEMA/HTCC10% demonstrated 79.98% cell viability in the MTT assay, surpassing the 70% threshold for noncytotoxicity according to ISO 10993.^60,61^ These findings highlight the trade-off between antibacterial performance and cytotoxicity, emphasizing the need to optimize HTCC content to balance antimicrobial activity and biocompatibility.

### 3.7 *In Vitro* Papain Release

The cumulative release profiles of papain from PDMAEMA-based hydrogels incorporated with different concentrations of HTCC demonstrate the significant influence of the HTCC content on the release kinetics. In the absence of HTCC (Figure 7a), the hydrogel released approximately 60% of papain over 48 h, with the majority of the release occurring within the first 10 h. This behavior is characteristic of a highly hydrated hydrogel, where diffusion dominates the transport mechanism. Upon the incorporation of 5% HTCC (Figure 7b), the release reached nearly 90%, suggesting that a small amount of HTCC can enhance the release capacity. This may be attributed to increased hydrophilicity and possibly the higher swelling capacity of this sample, as demonstrated in Figure 2a, which may have facilitated the diffusion of papain molecules.

**Figure 7:**
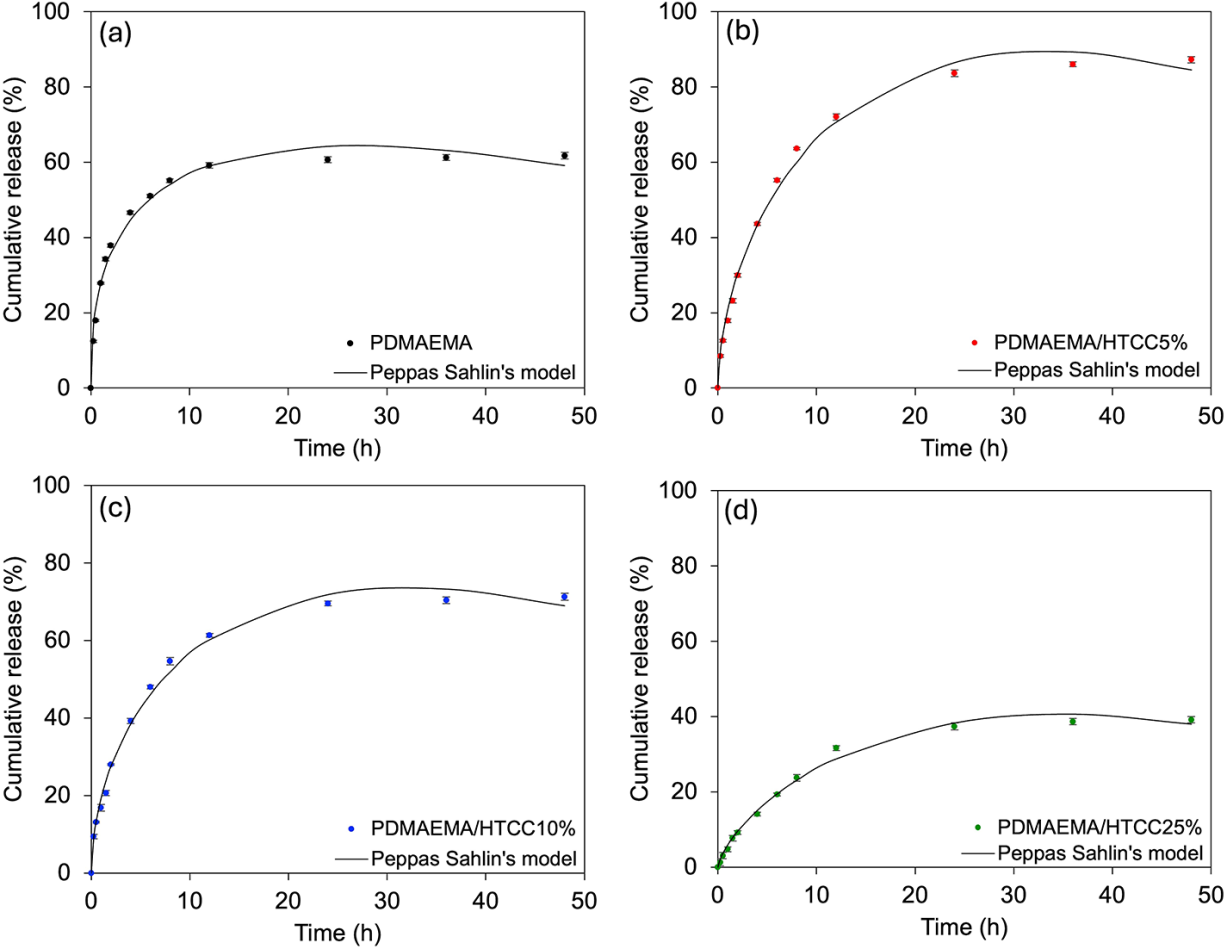
Papain release profiles from hydrogels composed of PDMAEMA and varying HTCC contents: (a) PDMAEMA, (b) PDMAEMA/HTCC5%, (c) PDMAEMA/HTCC10%, and (d) PDMAEMA/HTCC25%.

However, further increases in the HTCC content resulted in a gradual decrease in papain release. With 10% HTCC (Figure 7c), cumulative release reached about 75%, while at 25% HTCC (Figure 7d), the release dropped to approximately 40% over the same period. This inverse correlation between HTCC content and release efficiency is likely due to the formation of a denser network through electrostatic interactions between the cationic HTCC and the polymer matrix as well as stronger interactions with papain itself. Previously, SEM micrographs and pore size distribution analyses (Figure 2) indicated that increasing the HTCC content leads to a denser and less porous network. This compaction is particularly evident at 25% HTCC, where the hydrogel exhibits markedly smaller and less interconnected pores. Such structural densification correlates strongly with the reduced equilibrium swelling observed in Figure 2a. At higher HTCC levels, interactions between the hydroxyl groups of chitosan and the tertiary amines of PDMAEMA become more dominant,^43,44^ resulting in smaller pore formation and decreased water uptake, consequently reducing papain release. This inverse correlation between the increase in HTCC content and the reduction in the hydrogel’s swelling capacity, which in turn modulates drug release, is consistent with observations from other semi-IPN systems. Analogously, Guo et al. (2007)^17^ also reported that an increase in carboxymethyl chitosan content in PDMAEMA-based hydrogels led to a decrease in the swelling degree and, consequently, a lower drug release rate.

Figure 7 also indicates the fit of experimental data with the Peppas–Sahlin model. This classical model allows for the separation of Fickian diffusion and polymer relaxation contributions.^28^ Table 4 indicates that all four formulations exhibited strong fits to the model, with *R* ^2^ values above 0.99, indicating that this model is more appropriate for describing these systems than the Korsmeyer–Peppas model. In all cases, the contribution of Fickian diffusion (*k*_1_) was substantially greater than that of relaxation-controlled transport (*k*_2_), which was consistently negative and small in magnitude. This suggests that the release process is predominantly diffusion-controlled and that matrix relaxation plays a minor or possibly retarding role in papain transport.^62^ Interestingly, considering the Peppas–Sahlin model (Table 4), the *k*_1_ values decreased systematically with increasing HTCC content, from 31.885 (PDMAEMA) to 6.135 (HTCC25%), reflecting the progressive reduction in diffusion rate. Simultaneously, the *k*_2_ values became less negative, indicating a diminishing influence of the polymer relaxation. The ratio of |*k*_2_/*k*_1_| decreased across the series, reinforcing the interpretation that diffusion became increasingly dominant as the HTCC concentration increased. However, this impressive drop in *k*_1_ also suggests a more hindered regime, in which diffusion itself became limited due to the compact network and reduced water uptake at high HTCC concentrations, corroborating with the previous physicochemical and morphological discussions.

**Table 4:** Estimated Kinetic Parameters of Papain Release from PDMAEMA/HTCC Hydrogels according to Korsmeyer–Peppas and Peppas–Sahlin Models

	Korsmeyer–Peppas			Peppas–Sahlin			
	*k*	*n*	*R* ^2^	*k*_1_	*k*_2_	*m*	***R*** ^2^
PDMAEMA	30.635	0.212	0.955	31.885	–3.951	0.425	0.990
PDMAEMA/HTCC5%	25.849	0.345	0.971	22.087	–1.363	0.595	0.997
PDMAEMA/HTCC10%	23.657	0.317	0.968	20.912	–1.487	0.561	0.997
PDMAEMA/HTCC25%	8.382	0.432	0.970	6.135	–0.232	0.726	0.997

While this study focused on evaluating papain release under physiological conditions (pH 7.4, 37 °C) to simulate a relevant wound-healing environment, the dual-responsive nature of the PDMAEMA/HTCC hydrogel system suggests that its release behavior may also vary under different pH and temperature conditions. Given the distinct swelling patterns observed across a broad pH and thermal range, future investigations should examine how these stimuli influence the release kinetics of papain and other therapeutic agents, particularly in biomedical contexts, where variations in pH are physiologically meaningful. Such studies would offer deeper insights into the hydrogel’s potential for site-specific or condition-triggered delivery in dynamic physiological or pathological environments, thereby broadening its range of applications.

## 4 Conclusions

The systematic incorporation of HTCC into PDMAEMA-based semi-IPN hydrogels unveiled a clear structure–function relationship with network density and bioactivity tightly regulated by HTCC content. While lower HTCC levels (5%) favored swelling and rapid papain release, higher concentrations introduced a tighter network architecture that slowed diffusion and enhanced the antibacterial performance. Notably, the formulation containing 25% HTCC emerged as the most suitable candidate for wound-healing applications: although it showed reduced swelling (168.23%) and lower diffusion constant (*k*_1_ = 6.14 h^–*m*^), it provided a highly desirable combination of sustained papain release, nearly complete bacterial inhibition, and acceptable cytocompatibility. Despite its attenuated pH and thermoresponsiveness compared to lower HTCC formulations, these features may be of secondary importance in chronic wound environments, where prolonged antimicrobial action and biocompatibility are prioritized. These results point to a critical trade-off between hydration capacity and functional bioactivity, highlighting PDMAEMA/HTCC25% as a rationally balanced and tunable platform for controlled papain release in environments demanding both responsiveness and antibacterial action. Therefore, this work underscores the potential of semi-IPN design as a versatile approach for engineering smart hydrogels where delivery rate, structure, and antimicrobial defense can be co-optimized.
